# Supplementary material for: A New SNP-Based Genotyping Method for C. psittaci: Application to Field Samples for Quick Identification
Source: Microorganisms. 2021 Mar 17;9(3):625. doi: 10.3390/microorganisms9030625 (PMC8002925; doi:10.3390/microorganisms9030625)
Supplement: Supplementary file 1 [file microorganisms-09-00625-s001.zip › microorganisms-1114518-supplementary.pdf]

microorganisms

Supplementary Materials:

HRM group	strain/sample	ID	Host	Country	*omp* A Genotype	MLST	Reference
**I_Psittacine**	reference strains	6BC-04DC45	psittacine	USA	A	24	Sachse et al., 2008
		Loth	pigeon	France	A	-	unpublished data
		VS1	psittacine	USA	A	24	Sachse et al., 2008
	field strains with typing data	84-6461	psittacine	France	A	-	unpublished data
		84-8471/1	parrot	France	A	24	Sachse et al., 2009
		84-9462	unknown	France	A	24	Sachse et al., 2009
		85-1173	parrot	France	A	-	Sachse et al., 2009
		85-12098	parrot	France	A	-	Sachse et al., 2009
		86-0191	psittacine	France	A	-	Sachse et al., 2009
		86-10703	unknown	France	A	-	Sachse et al., 2009
		86-14356	parrot	France	A	-	Sachse et al., 2009
		86-3389	parakeet	France	A	-	Sachse et al., 2009
		87-13654	unknown	France	A	-	Sachse et al., 2009
		88-2014	parakeet	France	A	-	Sachse et al., 2009
		88-5558	parrot	France	A	-	Sachse et al., 2009
		88-5821	parakeet	France	A	-	Sachse et al., 2009
		88-8795	parakeet	France	A	-	Sachse et al., 2009
		89-2930	parakeet	France	A	-	Sachse et al., 2009
		90-0057	parakeet	France	A	-	Sachse et al., 2009
		90-0475	parrot	France	A	-	Sachse et al., 2009
		90-10445	parrot	France	A	-	Sachse et al., 2009
		90-11404	parrot	France	A	54	Sachse et al., 2009
		90-12937	parrot	France	A	-	Sachse et al., 2009
		90-4862	psittacine	France	A	24	unpublished data
		91-14273	psittacine	France	A	-	unpublished data
		91-5189	psittacine	France	A	24	unpublished data
		91-6047	parrot	France	A	-	Sachse et al., 2009
		95-1334	parrot	France	A	-	Sachse et al., 2009
		97-5075	psittacine	France	A	-	unpublished data
		97-6475	parakeet	France	A	-	Sachse et al., 2009
		98-7627	parrot	France	A	-	Sachse et al., 2009
		99-0182	parrot	France	A	-	Sachse et al., 2009
		99-0923	parakeet	France	A	-	Sachse et al., 2009
		99-8157/1	parrot	France	A	-	Sachse et al., 2009
		00-0151	psittacine	France	A	-	unpublished data
		00-0476	psittacine	France	A	-	unpublished data
		00-1176	psittacine	France	A	-	unpublished data
		00-1268	psittacine	France	A	-	unpublished data
		00-1750	psittacine	France	A	-	unpublished data
		05-0949	budgerigar	France	A	-	Sachse et al., 2009
		05-4098	parrot	France	A	-	Sachse et al., 2009
		06-0372	psittacine	France	A	-	unpublished data
		06-0852	psittacine	France	A	-	unpublished data
		C6/98	rabbit	Germany	A	24	this study
		01DC11	swine	Germany	A	24	this study
		03DC29	psittacine	Germany	A	24	this study
		03DC35	cattle	Germany	A	24	this study
		04DC42	laboratory rat	Germany	A	24	this study
		04DC46	tick	Germany	A	24	this study
	field strains without typing data	96-6274	psittacine	France	-	-	
		96-12328	psittacine	France	-	-	
		96-12742	psittacine	France	-	-	
		97-822	psittacine	France	-	-	
		97-9244	psittacine	France	-	-	
		99-0313	psittacine	France	-	-	
		99-1394	psittacine	France	-	-	
		99-8888	psittacine	France	-	-	
		00-4462	psittacine	France	-	-	
		03-3227	psittacine	France	-	-	
		04-2668	psittacine	France	-	-	
		10-0485	psittacine	France	-	-	
		10-1735	psittacine	France	-	-	
	clinical samples	10-1735*	psittacine	France	-	-	
		17-10114	psittacine	France	-	-	
		17-10090	psittacine	France	-	-	
**II_Duck**	reference strain	GR9	duck	Europe	C	28	Sachse et al., 2008
	field strains with typing data	94-2306	duck	France	EB	27	Sachse et al., 2009
		05-4325	duck	France	EB	28	Sachse et al., 2009
		05-4461	duck	France	C	28	Sachse et al., 2009
		06-859	duck	France	EB	-	Sachse et al., 2009
		06-871	duck	France	EB	-	Sachse et al., 2009
		06-881	duck	France	EB	28	Sachse et al., 2009
		06-889	duck	France	EB	-	Sachse et al., 2009
		06-1683	human	France	EB	28	Sachse et al., 2009
		10-1398/28	ibis	France	C	28	Vorimore et al., 2013
		10-1400	duck	France	EB	-	unpublished data (identical to 06-859)
		C1/97	sheep	Germany	C	-	Sachse et al., 2009
		07-1391	poultry	France	-	28	this study
		08-2626_L3	duck	France	-	28	this study
		08-2626_L4	duck	France	-	28	this study
	field strains without typing data	04-5006	duck	France	-	-	
		05-553/17	duck	France	-	-	
		07-2962	duck	France	-	-	
		08-2850	duck	France	-	-	
		10-1393	duck	France	-	-	
	clinical samples	15-46D/8	mute swan	Poland	-	-	Szymańska-Czerwińska et al., 2017a
		15-53D/8	mute swan	Poland	-	-	Szymańska-Czerwińska et al., 2017a
		15-41/8	goose	Poland	-	-	
		15-63/3	duck	Poland	-	-	Szymańska-Czerwińska et al., 2017b
		16-1264_MJC	human	France	-	-	
		16-1264_JL1	human	France	-	-	
		16-1264_JL2	human	France	-	-	
		17-5203	human	France	-	-	
		2008_A	human	France	-	-	
		2009_A	human	France	-	-	
		20-3954_C054	human	France	-	-	
**III_Pigeon**	reference strains	CP3	pigeon	USA	B	27	Sachse et al., 2008
		Cal10	ferret	USA	E	35	Sachse et al., 2008
		MN	human	USA	E		Sachse et al., 2008
	field strains with typing data	90-12617	pigeon	France	B	55	Sachse et al., 2009
		91-6568	dove	France	B	-	Sachse et al., 2009
		91-12516	pigeon	France	B	-	Sachse et al., 2009
		89-13210	pigeon	France	E	35	Sachse et al., 2009
		91-5983	psittacine	France	E	-	Sachse et al., 2009
		05-4036	pigeon	France	E	-	Sachse et al., 2009
		09-295_JF5	pigeon	France	B	-	Gasparini et al., 2011
		09-295_JF8	pigeon	France	B	-	Gasparini et al., 2011
		09-295_J9	pigeon	France	B	-	Gasparini et al., 2011
		09-295_MB32	pigeon	France	B	-	Gasparini et al., 2011
		09-295_MB33	pigeon	France	B	-	Gasparini et al., 2011
		09-336_FA30634	pigeon	France	B	-	Gasparini et al., 2011
		09-487_T13	pigeon	France	B	-	Gasparini et al., 2011
		09-489_LP5	pigeon	France	B	-	Gasparini et al., 2011
		09-489_LP7	pigeon	France	B	-	Gasparini et al., 2011
		09-489_Mon5	pigeon	France	B	-	Gasparini et al., 2011
		09-489_Mon13	pigeon	France	B	-	Gasparini et al., 2011
		09-496_FA32303	pigeon	France	B	-	Gasparini et al., 2011
		09-496_FA32311	pigeon	France	B	-	Gasparini et al., 2011
		09-544_Van14	pigeon	France	B	-	Gasparini et al., 2011
		09-589_S10	pigeon	France	B	-	Gasparini et al., 2011
		09-928	pigeon	France	B	-	Gasparini et al., 2011
		10-743_SC1	pigeon	France	B	-	Gasparini et al., 2011
		10-881_SC22	pigeon	France	B	-	Gasparini et al., 2011
		10-743_SC24	pigeon	France	B	-	Gasparini et al., 2011
		10-743_SC28	pigeon	France	B	-	Gasparini et al., 2011
		10-743_SC33	pigeon	France	B	-	Gasparini et al., 2011
		10-743_SC42	pigeon	France	B	-	Gasparini et al., 2011
		10-881_SC42	pigeon	France	B	-	Gasparini et al., 2011
		10-883_EL27	pigeon	France	B	-	Gasparini et al., 2011
		10-1048_Bat16	pigeon	France	B	-	Gasparini et al., 2011
		01DC12	swine	Germany	E	-	this study
		03DC32	pigeon	Germany	B	-	this study
		09DC75	pigeon	Germany	B	-	this study
		11DC94	pigeon	Germany	B	-	this study
	field strains without typing data	10-2168	pigeon	France	-	-	
	clinical samples	15-8D/13	hooded crow	Poland	-	-	Szymańska-Czerwińska et al., 2017a
		15-57D/2	black-headed	Poland	-	-	Szymańska-Czerwińska et al., 2017a
		10-743_SC1*	pigeon	France	-	-	
		10-743_SC22*	pigeon	France	-	-	
		10-743_SC24*	pigeon	France	-	-	
		10-743_SC28*	pigeon	France	-	-	
		10-743_SC33*	pigeon	France	-	-	
		10-2168*	pigeon	France	-	-	
		16-1264_VE	human	France	-	-	
		20-1105_A036	human	France	-	-	
		20-1105_A039	human	France	-	-	
**IV_Turkey**	reference strains	NJ1	turkey	USA	D	43	Sachse et al., 2008
		TT3	turkey	USA	D	-	
**V_Mat116**	field strain with typing data	99DC05	horse	Germany	Mat116	218	Henning et al., 2000
	clinical samples	20-1105_A041	human	France	-	218	this study
		19-5617_I078_G2662	human	Sweeden	-	218	this study
		19-5617_I080_K12016	human	Sweeden	-	218	this study
**VI_M56**	reference strain	M56-07DC57	muskrat	Canada	H/M56	31	Sachse et al., 2008
	field strain with typing data	16DC111	cattle	Germany	M56	-	this study
	clinical samples	12-2090_P088	ruminant	France	M56	-	this study
		12-1950_M074	cattle	France	M56	-	this study
**VII_VS225**	reference strain	VS225	psittacine	USA	F/VS225	204	Sachse et al., 2008
**VIII_WC**	reference strain	WC-07DC58	cattle	-	G/WC	32	Sachse et al., 2008
	field strain with typing data	Ful127	fulmar	Faroe Islands	-	197	

* linked to a field strain

Supplementary Data 1. PCR-HRM clustering of reference strains, field strains and clinical samples from human and animal origin. Available genotype and/or MLST results are indicated.

Strain	Accession number
Chlamydia psittaci 01DC11	GCA_000415525.1
Chlamydia psittaci 01DC12	GCA_000317995.1
Chlamydia psittaci 02DC14	GCA_000417565.1
Chlamydia psittaci 02DC15	NC_019391.1
Chlamydia psittaci 02DC16	GCA_000415565.2
Chlamydia psittaci 02DC18	GCA_000415585.1
Chlamydia psittaci 02DC21	GCA_000415605.1
Chlamydia psittaci 02DC22	GCA_000415625.1
Chlamydia psittaci 02DC23	GCA_000415645.1
Chlamydia psittaci 02DC24	GCA_000417585.1
Chlamydia psittaci 03DC29	GCA_000415665.1
Chlamydia psittaci 03DC35	GCA_000415825.1
Chlamydia psittaci 04DC42	GCA_000415685.2
Chlamydia psittaci 06-1683	GCA_000415845.1
Chlamydia psittaci 08-2626 L3	GCA_000417825.1
Chlamydia psittaci 08DC60	NC_017290.1
Chlamydia psittaci 09DC77	GCA_000415725.1
Chlamydia psittaci 09DC78	GCA_000415745.1
Chlamydia psittaci 09DC79	GCA_000417605.1
Chlamydia psittaci 09DC80	GCA_000415765.1
chlamydia psittaci 6BC	NC_017287.1
Chlamydia psittaci 84/55	CP003790.1
Chlamydia psittaci 84-8471/1	GCA_000417845.2
Chlamydia psittaci 99DC5	GCA_000415785.1
Chlamydia psittaci AMK	CP047319
Chlamydia psittaci C1/97	GCA_000417655.1
Chlamydia psittaci C19/98	NC_017291.1
Chlamydia psittaci C6/98	GCA_000417675.1
Chlamydia psittaci Cal10	GCA_000204175.2
Chlamydia psittaci CB3	GCA_000687415.1
Chlamydia psittaci CB7	GCA_000687395.1
Chlamydia psittaci CP3	CP003797.1
Chlamydia psittaci CR009	GCA_001714575.1
Chlamydia psittaci DD34	GCA_001401465.1
Chlamydia psittaci Fa An	GCA_001714595.1
Chlamydia psittaci Fr Da	GCA_001714565.1
Chlamydia psittaci Ful 127	NZ_CP033059.1
Chlamydia psittaci GR9	NC_018620.1
Chlamydia psittaci HJ	GCA_000786005.2
Chlamydia psittaci Ho Re lower	GCA_001735985.1
Chlamydia psittaci Ho Re upper	GCA_001736055.1
Chlamydia psittaci Horse pl	CP025423
Chlamydia psittaci M56	NC_018623.1
Chlamydia psittaci Mat116	CP002744.1
Chlamydia psittaci MN	NC_018627.1
Chlamydia psittaci NJ1	NC_018626.1
Chlamydia psittaci Po An	GCA_001736045.1
Chlamydia psittaci RD1	FQ482149.1
Chlamydia psittaci Rostinovo-70	CP041038
Chlamydia psittaci str. Frances	GCA_001401485.1
Chlamydia psittaci UGA	GCA_001563055.1
Chlamydia psittaci VS225	CP003793.1
Chlamydia psittaci WC	NC_018624.1
Chlamydia psittaci WS/RT/E30	NC_018622.1
Chlamydia psittaci Zo Pa	GCA_001714585.1

Supplementary Data 2. NCBI accession numbers of the 55 *C. psittaci* strains used in this study.

		**SNP for each group**											
**SNP No.**	**Associated Group**	Group I (Psittacine)	Group II (Duck)	Group III (Pigeon)	Group IV (Turkey)	Group V (M56)	Group VI (Mat116)	Group VII (VS225)	Group VIII (WC)	**Allele of the respective subtype**	**Tm values (°C)**	**Allele of the other subtypes**	**Tm values (°C)**
**1**	Group I_Psittacine	**T**	C	C	C	C	C	C	C	T	76,36 ± 0,01	C	76,65 ± 0,02
**2**	Group II_Duck	T	**C**	T	T	T	T	T	T	C	76,30 ± 0,07	T	75,79 ± 0,09
**3**	Group III_Pigeon	A	A	**G**	A	A	A	A	A	G	76,11 ± 0,03	A	75,50 ± 0,04
**4**	Group IV_Turkey	C	C	C	**T**	C	C	C	C	T	75,19 ± 0,09	C	75,82 ± 0,11
**5**	Group V_M56	C	C	C	C	**T**	C	C	C	T	74,83 ± 0,12	C	75,29 ± 0,12
**6**	Group VI_Mat116	T	T	T	T	T	**C**	T	T	C	76,16 ± 0,13	T	75,58 ± 0,09
**7**	Group VII_VS225	A	A	A	A	A	A	**G**	A	G	76,298 ± 0,10	A	75,358 ± 0,09
**8**	Group VIII_WC	G	G	G	G	G	G	G	**A**	A	76,29 ± 0,07	G	77,12 ± 0,07

Supplementary Data 3. Melting temperature (Tm) values for each SNP. Values were determined for the respective controls tested 6 times.

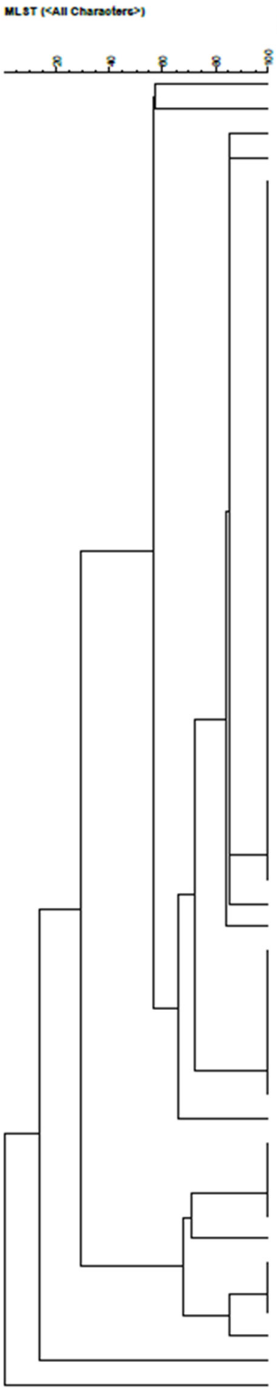

MLST

gatA	oppA	hflX	gidA	enoA	hemN	fumC	
12	13	18	13	68	9	10	Chlamydia psittaci VS225
12	13	13	13	13	13	10	Chlamydia psittaci WC
38	13	11	13	13	9	10	Chlamydia psittaci Mat116
74	13	11	13	13	9	10	Chlamydia psittaci Zo Pa
10	13	11	13	13	9	10	Chlamydia psittaci 01DC11
10	13	11	13	13	9	10	Chlamydia psittaci 02DC14
10	13	11	13	13	9	10	Chlamydia psittaci 02DC15
10	13	11	13	13	9	10	Chlamydia psittaci 02DC16
10	13	11	13	13	9	10	Chlamydia psittaci 02DC18
10	13	11	13	13	9	10	Chlamydia psittaci 02DC21
10	13	11	13	13	9	10	Chlamydia psittaci 02DC22
10	13	11	13	13	9	10	Chlamydia psittaci 02DC23
10	13	11	13	13	9	10	Chlamydia psittaci 02DC24
10	13	11	13	13	9	10	Chlamydia psittaci 03DC29
10	13	11	13	13	9	10	Chlamydia psittaci 03DC35
10	13	11	13	13	9	10	Chlamydia psittaci 04DC42
10	13	11	13	13	9	10	Chlamydia psittaci 08DC60
10	13	11	13	13	9	10	Chlamydia psittaci 6BC
10	13	11	13	13	9	10	Chlamydia psittaci 84-8471/1
10	13	11	13	13	9	10	Chlamydia psittaci C19/98
10	13	11	13	13	9	10	Chlamydia psittaci C6/98
10	13	11	13	13	9	10	Chlamydia psittaci Cal10
10	13	11	13	13	9	10	Chlamydia psittaci CB3
10	13	11	13	13	9	10	Chlamydia psittaci CB7
10	13	11	13	13	9	10	Chlamydia psittaci CR009
10	13	11	13	13	9	10	Chlamydia psittaci DD34
10	13	11	13	13	9	10	Chlamydia psittaci Fa An
10	13	11	13	13	9	10	Chlamydia psittaci Fr Da
10	13	11	13	13	9	10	Chlamydia psittaci Ho Re lower
10	13	11	13	13	9	10	Chlamydia psittaci Ho Re uppe
10	13	11	13	13	9	10	Chlamydia psittaci Horse_pl
10	13	11	13	13	9	10	Chlamydia psittaci Po An
10	13	11	13	13	9	10	Chlamydia psittaci RD1
10	13	11	13	13	9	10	Chlamydia psittaci UGA
10	13	11	13	71	9	10	Chlamydia psittaci 99DC5
10	13	61	13	13	9	10	Chlamydia psittaci Ful 127
12	13	11	13	13	9	12	Chlamydia psittaci 06-1683
12	13	11	13	13	9	12	Chlamydia psittaci 08-2626_L3
12	13	11	13	13	9	12	Chlamydia psittaci AMK
	13	11	13	13	9	12	Chlamydia psittaci C1/97
12	13	11	13	13	9	12	Chlamydia psittaci GR9
12	13	11	13	13	9	12	Chlamydia psittaci Rostinovo-7
12	13	11	13	13	9	12	Chlamydia psittaci WS/RT/E30
10	13	18	13	13	11	10	Chlamydia psittaci 84/55
11	14	15	13	13	9	13	Chlamydia psittaci 09DC77
11	14	15	13	13	9	13	Chlamydia psittaci 09DC78
11	14	15	13	13	9	13	Chlamydia psittaci 09DC79
11	14	15	13	13	9	13	Chlamydia psittaci 09DC80
11	14	15	14	13	9	11	Chlamydia psittaci CP3
11	14	15	14	13	11	13	Chlamydia psittaci HJ
11	14	15	14	13	11	13	Chlamydia psittaci MN
11	14	15	14	13	11	13	Chlamydia psittaci str. Frances
11	14	11	14	13	11	13	Chlamydia psittaci 01DC12
17	17	17	18	13	12	16	Chlamydia psittaci M56
15	16	13	16	15	10	15	Chlamydia psittaci NJ1

Supplementary Data 4. MLST-based tree determined from ST of the 55 *C. psittaci* strains used for the SNP determination. The tree was built using BioNumerics software with the parameter "categorical values" to calculate the similarity matrix and UPGMA to reconstruct the tree.
